# Advances in the study of reversing tumor drug resistance by targeting cancer-associated fibroblasts with nanomaterials

**DOI:** 10.3389/fimmu.2025.1647988

**Published:** 2025-11-19

**Authors:** Xiaozhuo Gao, Lili Sun, Tianming Li, Pengfei Li, Chang Liu, Qiang Li, Yanmei Zhu

**Affiliations:** 1 Department of Pathology, Affiliated Cancer Hospital of Dalian University of Technology (Liaoning Cancer Hospital and Institute, Cancer Hospital of China Medical University), Shenyang, China; 2 Department of Thoracic Surgery, Affiliated Cancer Hospital of Dalian University of Technology (Liaoning Cancer Hospital and Institute, Cancer Hospital of China Medical University), Shenyang, China; 3 Department of Central Laboratory, Affiliated Cancer Hospital of Dalian University of Technology (Liaoning Cancer Hospital and Institute, Cancer Hospital of China Medical University), Shenyang, China

**Keywords:** nanomaterials, cancer-associated fibroblasts, tumor microenvironment, tumor drug resistance, reverse tumor drug

## Abstract

Cancer-associated fibroblasts (CAFs) are key components of the tumor microenvironment (TME) which promote drug resistance by remodeling the extracellular matrix, generating an immunosuppressive microenvironment, and activating metabolic signaling pathways. Nanomaterials provide an effective method for specifically targeting CAF-mediated drug resistance because of their unique targeted delivery capabilities, responsive release characteristics, and multifunctional integration. Here, we describe the mechanisms underlying the role of CAFs in drug resistance. The types of materials used and design principles are described, and examples of the application of nanomaterials for targeting CAFs are provided. Current challenges and future directions of nanomaterials targeting CAFs for reversing tumor drug resistance are also discussed to provide theoretical support for the development of effective nanotherapies aimed at reversing drug resistance in cancer.

## 1 Introduction

Tumor drug resistance is closely related to the dynamic regulation of the tumor microenvironment (TME) (1). The TME includes tumor cells and surrounding stromal cells, immune cells, and secreted factors, which together affect tumor growth, invasion and the response to treatment (1). Dynamic changes in the TME can promote tumor growth, hinder immune surveillance, and lead to treatment resistance in targeted therapy and immunotherapy (2). Cancer-associated fibroblasts (CAFs) are a heterogeneous group of cells that are abnormally activated in the tumor stroma and play a crucial role in the TME. Because of their heterogeneity, CAFs confer a drug resistance phenotype in tumor cells through many mechanisms, such as by acting as a physical barrier, affecting signaling pathways, or by affecting metabolic support and the immune microenvironment. Complex CAF-mediated drug resistance networks are challenging for traditional single-target therapy strategies.

Nanomaterials provide an innovative solution to this problem by virtue of their unique size effect, surface modification and functional integration advantages. Recent reviews highlight the critical role of CAFs in mediating tumor drug resistance and the promising applications of nanomaterials in targeting CAFs. Nanotherapeutic strategies include surface modification with CAF-specific ligands (e.g., FAP antibodies, peptide conjugates) for precision delivery, stimuli-responsive systems (pH/enzyme-sensitive nanoparticles) for controlled drug release, and multimodal platforms co-loading CAF inhibitors and chemotherapeutics (3).

Emerging evidence demonstrates that nanomaterials can normalize the tumor stroma, enhance drug penetration, and synergize with immunotherapy by reversing CAF-mediated immune suppression (4). Nanomaterials possess significant advantages, such as improved pharmacokinetics and biodistribution, long circulation, targeting, and controlled release. Improved pharmacokinetics/biodistribution: Nanomaterials (e.g., PEGylated polymeric micelles, lipid nanoparticles) overcome small-molecule limitations (short circulation, non-specific accumulation). They leverage the enhanced permeability and retention(EPR) effect to accumulate in CAF-rich tumor stroma, achieving a 40% higher tumor-to-plasma concentration ratio than free drugs (5). Long circulation: PEGylation reduces reticuloendothelial system(RES) clearance, extending nanocarrier circulation to 10–24 h (vs. 1–2 h for free drugs). This prolongs exposure, increasing tumor targeting chances (5, 6). Precision targeting: Ligand-modified nanocarriers (e.g., FAP antibody, RGD peptide) bind CAF markers (FAP, α-SMA), cutting liver/spleen accumulation by 50% vs. non-targeted carriers and avoiding normal fibroblast damage (5, 7). Controlled release: Stimuli-responsive systems (pH/ROS-sensitive micelles) release drugs (doxorubicin, TGF-β inhibitors) over 24–48 h in CAF-rich TME, maintaining effective concentrations to inhibit CAF activation/ECM synthesis (5, 7).

## 2 Biological characteristics of CAFs and mechanisms of CAF-mediated tumor drug resistance

### 2.1 Origin and heterogeneity of CAFs

CAFs are a group of abnormally activated fibroblasts in the TME that arise from a diversity of cells, including tissue-resident fibroblasts (8), mesenchymal stem cells (9, 10), epithelial cells (through epithelial-mesenchymal transition, EMT) (11), endothelial cells (through endothelial-mesenchymal transition), adipocytes and perivascular cells (12, 13). These precursor cells are transformed into activated CAFs in response to specific signaling factors in the TME, such as transforming growth factor (TGF)-β, stromal cell-derived factor 1, and interleukin (IL-6) (14, 15).

CAFs constitute a heterogeneous cell population that is divided into subtypes according to phenotype and function. Myofibroblast-like CAFs (myCAFs), which express high levels of α-SMA and collagen fibers, regulate extracellular matrix (ECM) remodeling and tumor mechanical stiffness (16, 17); they play an important role in pancreatic cancer, breast cancer, and other stromal tumors. Inflammatory CAFs (iCAFs) secrete pro-inflammatory factors such as IL-6 and CXCL1, activate the JAK/STAT3 pathway, recruit immunosuppressive cells such as MDSCs and M2 macrophages, and form an immune escape microenvironment (18). Antigen-presenting CAFs (apCAFs) express high levels of MHCII, which interacts with T cells and is involved in the responsiveness to immunotherapy (19). Lipid-rich CAFs, which promote tumor growth by transporting lipids, are induced in SETD2-deficient pancreatic cancer cells (20). SETD2 is a histone lysine methyltransferase whose deficiency leads to metabolic reprogramming of tumor cells and immune escape (21). Vascular CAFs localize predominantly to the core area of tumors and promote angiogenesis by secreting various factors such as vascular endothelial growth factor to support tumor growth and spread (22, 23).

### 2.2 Key mechanisms of CAF-mediated tumor drug resistance

CAFs are important components of the TME that promote drug resistance through multi-dimensional mechanisms, including the formation of physical barriers, activation of drug resistance pathways, activation of signaling pathways promoting dryness and invasion, generation of an immune escape microenvironment, maintenance of the drug resistance phenotype, and tumor cell resistance memory.

Physical barriers and drug delivery obstacles: CAFs secrete a large number of ECM components such as collagen, thereby increasing the stiffness of the matrix by regulating the composition and structure of the ECM. This results in the formation of a physical barrier that limits the penetration of chemotherapeutic drugs, which is one of the key causes of chemotherapy resistance in cancer (24, 25). CAF-secreted collagen decreases the concentration of doxorubicin in the tumor core; the resulting increase in matrix stiffness hinders drug diffusion, resulting in partial survival of tumor cells and induction of drug resistance (26, 27).

Metabolic reprogramming and energy support: lipid-rich CAFs provide fatty acids to tumor cells via ATP-binding cassette subfamily A member 8a (ABCA8a), enhancing their mitochondrial function, anti-metabolic drugs and mitochondrial targeting drugs (28). iCAFs and myCAFs upregulate GLUT1 and LDH, transport lactate to tumor cells through MCT4 (29), activate the HIF-1α pathway, induce the expression of drug resistance genes such as MDR1, and increase drug resistance in tumor cells (30, 31). CAFs increase glutathione (GSH) levels in cancer cells through secretion mediators, decrease drug-induced reactive oxygen species production and DNA damage, and promote chemotherapy resistance (32).

Signaling pathway activation and tumor cell survival: hepatocyte growth factor (HGF) secreted by CAFs activates the c-Met receptor and restores PI3K/Akt and MAPK/ERK signaling, leading to epidermal growth factor (EGFR)-tyrosine kinase inhibitor (TKI) resistance (33). Combination treatment with c-Met inhibitors increases the efficacy of EGFR-TKIs, thereby inhibiting tumor cell growth and proliferation (34) and delaying relapse in drug-resistant tumors (35). C-X-C Motif Chemokine Ligand 12 (CXCL12) secreted by CAFs binds to tumor cell CXCR4, thereby activating downstream signaling that enhances cancer stem cell (CSC) drying and invasion, leading to tumor progression and metastasis (36–38). Drug resistance in CSCs is acquired through multiple mechanisms, including overexpression of molecules such as aldehyde dehydrogenase 1 (ALDH1) and ATP-binding cassette subfamily G member 2 (ABCG2). ALDH1 is an enzyme involved in cellular redox reactions, and its high activity is closely related to tumor invasiveness and drug resistance (39, 40). ABCG2 is a transmembrane protein that pumps chemotherapy drugs out of cells, thereby reducing their cytotoxicity (41, 42). CAFs promote CSC enrichment by activating the CXCL12/CXCR4 axis and increase the migratory ability and drug resistance of tumor cells by inducing EMT, further aggravating the malignant progression of tumors (43, 44).

Immunosuppressive microenvironment and immunotherapy resistance: iCAFs secrete CCL2 to recruit MDSCs and inhibit T cell activity by secreting multiple inhibitory factors in the TME (45). IL-6 secreted by iCAFs promotes M2 macrophage polarization and inhibits CD8 +T cell function through the inhibitory factor IL-10 (46, 47). Although apCAFs express MHCII, they often cannot activate T cells effectively because of the expression of co-inhibitory molecules (PD-L1, CTLA-4) or defects in antigen processing, and they may even induce immune tolerance. For example, the combination of PD-L1 and PD-1 can lead to T cell failure and inhibit immune responses (48, 49). ApCAFs may be deficient in antigen processing. Although some cell types can continue to synthesize MHCII molecules after activation, they lack effective antigen processing mechanisms, resulting in ineffective presentation of exogenous antigens (50). Hypoxic conditions in the TME may also help tumor cells evade immune surveillance by downregulating the expression of MHCI and MHCII to limit antigen presentation (51).

Epigenetic regulation and drug resistance memory: the involvement of CAFs in drug resistance is mediated by several mechanisms including epigenetic reprogramming (DNA methylation and histone modification) (52). For example, activation of the YAP/TAZ signaling pathway can promote tumor cell growth (53), whereas histone deacetylase (HDAC) inhibitors increase histone acetylation by inhibiting HDAC activity, thereby affecting epigenetic reprogramming of CAFs (YAP/TAZ signaling pathway) and interfering with their role in promoting drug resistance (54). However, this process may trigger a compensatory mechanism of CAFs (through YAP/TAZ activation to counteract drug action) and conserve the drug resistance-promoting function (55). These processes highlight the complexity of CAF drug resistance mechanisms.

The involvement of CAFs in drug resistance suggests that targeting CAFs could be an important strategy to improve the efficacy of cancer treatment.

## 3 Types and core strategies of nanomaterials targeting CAFs

Nanomaterials are optimal carriers for targeting CAFs because of their unique physicochemical properties and biocompatibility. Nanomaterials can be divided into organic nanomaterials and inorganic nanomaterials according to their chemical composition and functional properties. Organic nanomaterials are carbon-based molecules including lipid-based nanoparticles, polymer nanoparticles, and dendrimers. They are biocompatible and can be designed to specifically recognize and bind to CAFs, which increases the efficacy of drug delivery and optimizes the therapeutic effects (56). Inorganic nanomaterials do not contain carbon and can modify the CAF microenvironment through optical and magnetic effects, such as metal nanomaterials and carbon-based nanomaterials. They can be developed into effective tools for targeting CAFs due to their excellent stability and easily modified surface characteristics (57). Other types of nanomaterials include biological nanoparticles and hybrid nanomaterials. Nanomaterial-based CAF targeting strategies aim to modulate CAF function and reverse tumor drug resistance through multiple pathways, including clearing and killing CAFs, inhibiting CAF activation and reprogramming, and disrupting CAF function.Detailed information regarding the types and core strategies of CAFs-targeting nanomaterials is shown in **Table 1**.

**Table 1:** Core strategies for nanomaterials targeting CAFs.

Material type	Core strategy	Mechanism of action	Specific materials	Application effect	Reference(s)
Organic Nanomaterials	Precise Targeting of Surface Markers	Modify ligands (antibodies/aptamers) of CAFs surface markers for specific recognition and enrichment	FAP antibody-modified liposomes	Reduce tumor stroma density and enhance chemotherapeutic drug permeability	(53)
			Chitosan nanoparticles (encapsulating FAP siRNA)	Downregulate FAP expression and block CAFs-induced angiogenesis	(55)
	Responsive Release System Design	Achieve controlled drug release via TME-responsive acidity or enzyme concentration	Gelatin-encapsulated gold nanoparticles (modified with RGD peptide, responsive to MMP-2/9)	Enzyme-degrade the carrier to release doxorubicin, disrupt extracellular matrix (ECM), and enhance drug accumulation	(66–69)
	Gene and Metabolic Intervention	Deliver gene drugs or metabolic inhibitors to block CAFs functions	mPEG-PLGA nanoparticles loaded with baicalin	Inhibit TGF-β/Smad2/3 pathway, reduce CAFs activation and migration	(70–72)
			PLGA nanoparticles (loaded with glutaminase inhibitor CB-839, targeting PDGFR-β)	Reduce glutamine secretion and enhance the efficacy of cisplatin in gastric cancer	(77, 78)
	Synergistic Strategy	Co-load drugs or enable sequential release, integrating chemotherapy/immunotherapy/photothermal therapy	Liposomes co-loaded with nilotinib (CAFs inhibitor) and doxorubicin	Inhibit CAF-secreted IL-6 and enhance tumor cells' sensitivity to chemotherapy	(85, 86)
Inorganic Nanomaterials	Precise Targeting of Surface Markers	Combine physical properties of metal nanomaterials with targeting ligands	Bismuth ferrite harmonic nanoparticles (conjugated with FAP antibody)	MRI-guided targeting of pancreatic cancer CAFs, reduce stromal hardness by 40%, and enhance gemcitabine permeability by 2.5-fold	(54)
			Superparamagnetic iron oxide nanoparticles (SPIONs, targeting CAF apoptosis)	Induce CAF apoptosis via alternating magnetic field and inhibit CAF activity	(62, 63)
	Responsive Release System Design	Trigger drug release or photothermal effect via photo/magnetic responsiveness	pH-sensitive GO	Acidic environment triggers drug release, targeting CAF microenvironment	(65)
			Gold nanorods (modified with enzyme-responsive peptides)	Near-infrared light-triggered photothermal effect disrupts ECM and promotes drug penetration	(66)
	Gene and Metabolic Intervention	Deliver gene editing tools or metabolic disruptors	CRISPR-Cas9 nanocarriers (knockout of CXCR4 gene in CAFs)	Block CXCL12/CXCR4 axis, inhibit cancer stem cell (CSC) stemness and epithelial-mesenchymal transition (EMT)	(59–61)
	Synergistic Strategy	Photothermal effect combined with chemotherapy/immunotherapy	Liposome-AuNPs hybrid system (AuNPs photothermal effect + liposomal drug delivery)	Near-infrared light softens the stroma, enhances drug release and immune cell infiltration	(93–96)
Hybrid Nanomaterials	Surface Marker Precise Targeting + Responsive Release	Integrate multiple properties of organic-inorganic materials	FAP antibody- modified liposome-AuNPs complex	Target CAFs and combine photothermal effect for dual inhibition of CAF functions	(53, 93)
	Multimodal Synergistic Therapy	Magnetic targeting + hyperthermia + chemotherapy with multiple mechanisms	Superparamagnetic iron oxide (IONPs)-PLGA hybrid nanoparticles	Magnetic field enrichment in CAF regions, synergistic photothermal and chemotherapeutic killing of tumor cells	(97–102)
Biological Nanoparticles	Natural Source Targeted Delivery	Utilize biocompatible carriers (e.g., ferritin)	H-ferritin nanocages (loaded with Navitoclax, targeting FAP)	Specifically kill CAFs and enhance drug cytotoxicity	(56)

AuNPs, gold nanoparticles; CAFs, cancer-associated fibroblasts; CRISPR-Cas9, clustered regularly interspaced short palindromic repeats-CRISPR-associated protein 9; CSC, cancer stem cell; CXCR4, c-x-c chemokine receptor type 4; ECM, extracellular matrix; EMT, epithelial-mesenchymal transition; FAP, fibroblast activation protein; GO, graphene oxide; IONPs, iron oxide nanoparticles; MMP-2/9, matrix metalloproteinase-2/9; mPEG-PLGA, methoxy-Poly(Ethylene Glycol)-Poly(Lactic-co-Glycolic Acid); MRI, magnetic resonance imaging; siRNA, small interfering RNA; SPIONs, superparamagnetic iron oxide nanoparticles; TME, tumor microenvironment.

### 3.1 Precision targeting strategies for surface markers

**Figure 1:**
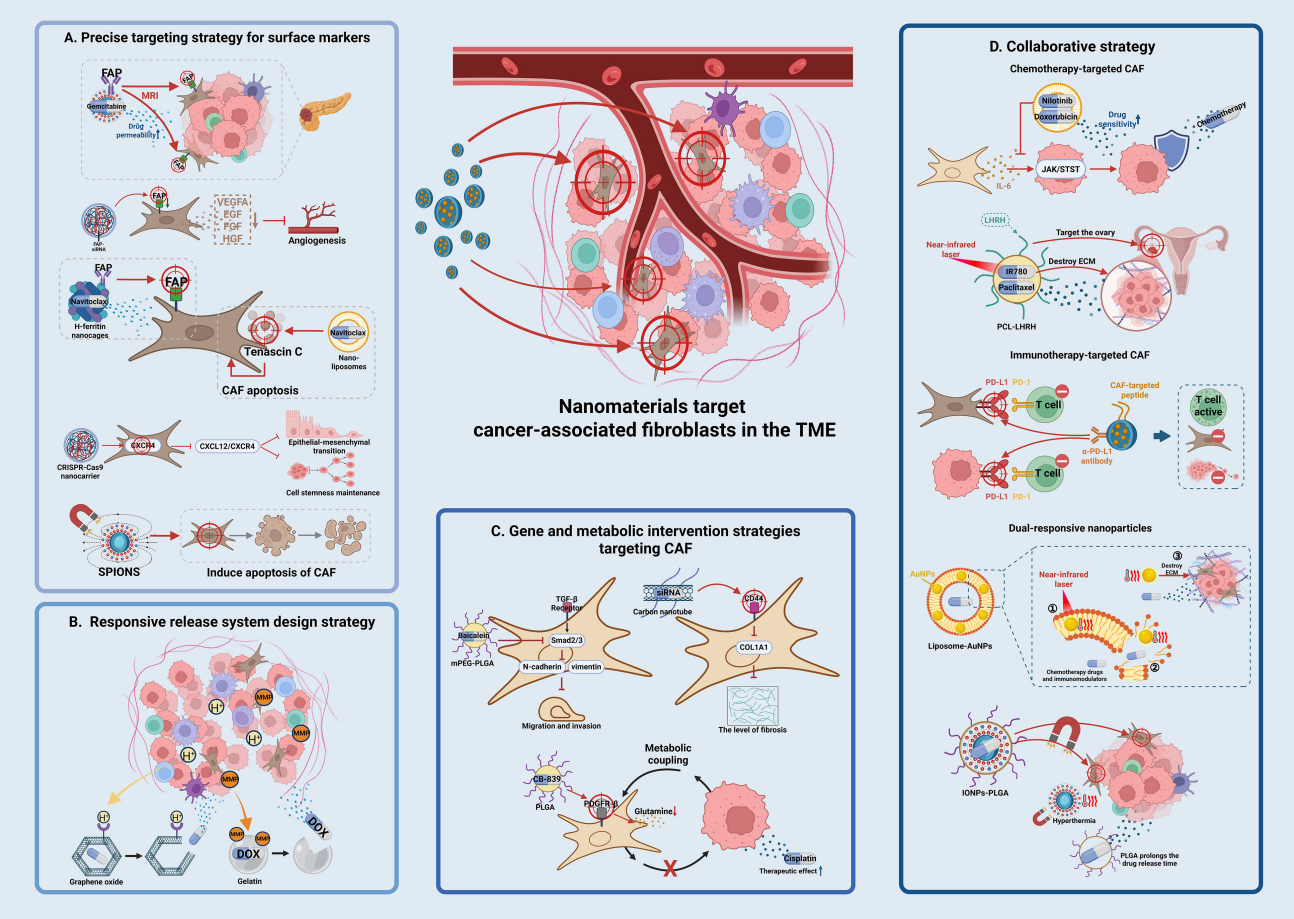
Schematic illustration of nanomaterial-based targeting strategies for CAFs in reversing tumor drug resistance. **(A)** Precision targeting strategies for surface markers nanomaterials are modified with ligands (e.g., antibodies, aptamers) to recognize CAF-specific surface markers such as FAP, α-SMA, or PDGFRβ. For example, FAP antibody-conjugated liposomes and bismuth ferrite nanoparticles enable specific binding to CAFs, reducing stromal density and enhancing drug penetration. CRISPR-Cas9 nanovectors are used to knock out CXCR4 in CAFs, disrupting the CXCL12/CXCR4 axis and inhibiting cancer stem cell properties. **(B)** Responsive release system design pH-sensitive nanoparticles (e.g., graphene oxide, GO) exploit the acidic tumor microenvironment (TME) to trigger drug release. Enzyme-responsive systems (e.g., MMP-2/9-degradable gelatin-coated gold nanoparticles) specifically degrade in the TME, releasing drugs like doxorubicin. These strategies enhance drug accumulation in tumors while minimizing systemic side effects. **(C)** Genetic and metabolic intervention strategies nanomaterials deliver gene drugs (e.g., siRNA, CRISPR-Cas9) or metabolic inhibitors to disrupt CAF functions. For instance, mPEG-PLGA nanoparticles loaded with baicalein inhibit TGF-β signaling, while PLGA nanoparticles carrying glutamine inhibitors (CB-839) target PDGFR-β to deplete energy supply for tumor cells. Carbon nanotubes with hyaluronic acid modification silence fibrosis-related genes (e.g., COL1A1) via CD44 receptor targeting. **(D)** Synergy strategies. Multifunctional nanosystems integrate chemotherapy, photothermal therapy, and immunotherapy. Liposomes co-loading nilotinib and doxorubicin suppress CAF-secreted IL-6 to enhance tumor cell sensitivity. Photothermal agents (e.g., IR780-loaded PCL nanoparticles) combined with near-infrared light disrupt the extracellular matrix, improving drug penetration. Dual-responsive nanoparticles sequentially release FAP inhibitors and chemotherapeutics, remodeling the TME and enhancing antitumor efficacy.

CAF surface-specific markers (such as FAP, α-SMA, and PDGFRβ) are used to accurately identify and enrich CAFs through nanomaterial surface modification using targeting ligands (antibodies, aptamers, peptides). FAP antibody-modified liposomes can specifically target CAFs and decrease the density of the tumor stroma, thereby increasing membrane permeability and the accumulation of chemotherapeutic drugs (58). Bismuth ferrite harmonic nanoparticles conjugated with FAP antibody can be designed to target pancreatic cancer CAFs and release chemotherapy drugs, decreasing tumor interstitial hardness by 40%, and resulting in a 2.5-fold increase in gemcitabine penetration (59). FAP siRNA encapsulated in chitosan nanoparticles can be enriched in the tumor stroma through mucosal adhesion, where it downregulates FAP expression in CAFs and blocks angiogenesis (60). Navitoclax loaded with H-ferritin nanocages targets CAFs via FAP antibody fragments, which markedly increases the cytotoxicity of the drug (61). Navitoclax loaded nanoliposomes can also achieve selective apoptosis of CAFs by specifically binding to the tenascin C protein secreted by CAFs (62). A CRISPR-Cas9 nanovector designed to knock out the CXCR4 gene in CAFs blocks the effect of the CXCL12/CXCR4 axis on maintaining stem cell dryness and EMT induction (63–65). Some nanoparticles have the ability to kill CAFs, such as superparamagnetic iron oxide nanoparticles (SPIONs) that induce CAF apoptosis under the action of alternating magnetic fields (66), effectively inhibiting CAF activity and altering the TME (67). The precise targeting strategy of nanomaterials against CAF surface markers is illustrated in **Figure 1A** and summarized in **Supplementary Table 1**.

Current marker-based strategies (targeting FAP, α-SMA, PDGFRβ) face two core challenges due to CAF heterogeneity: non-universal expression across pro-tumor CAF subtypes and off-target expression in normal stromal cells. For non-universality, FAP, a classic marker, is primarily enriched in myCAFs but barely expressed in iCAFs (which secrete IL-6) or apCAFs (which express MHCII) (28). FAP-targeted nanocarriers only reduce stromal stiffness but fail to inhibit iCAF-mediated immunosuppression, leaving a 45% gemcitabine resistance rate in tumor cells (68). For off-target risks, α-SMA (a myCAF marker) is highly conserved in normal vascular smooth muscle and cardiac fibroblasts, leading to nanocarrier accumulation in the liver/spleen (16). PDGFRβ, expressed in multiple CAF subtypes, also exists in bone marrow mesenchymal stem cells and pericytes, preventing distinction between pro-tumor CAFs and normal stromal cells (22). Thus, single-marker strategies are insufficient, and future designs should use single-cell omics-identified subtype-specific markers (e.g., IL-6R for iCAFs) for “combinatorial marker recognition” to enhance specificity (61).

### 3.2 Responsive release system design strategy

Nanoparticles can utilize the pH, enzyme concentration, and other characteristics of the TME to achieve controlled drug release and improve targeting. pH-sensitive nanoparticles can exploit the acidic nature of the TME to achieve efficient drug delivery and release, thereby overcoming chemotherapy resistance and reducing side effects (69). The pH response characteristics of graphene oxide (GO) are particularly important. pH-sensitive prodrug molecules on the GO surface can be modified to achieve controlled release under acidic conditions, which is important for the CAF microenvironment in cancer therapy (70).

Matrix metalloproteinases (MMP-2/9) that are expressed at high levels in CAFs are used for specific drug delivery and targeted therapy in the TME. This strategy involves coating gold nanoparticles with enzyme-degradable carriers, such as gelatin modified with RGD peptides, to target CAFs and disrupt the ECM (71, 72). Gelatin-based nanoparticles can achieve controlled drug release through the action of MMP-2/9. These nanoparticles can be specifically degraded in the TME to release loaded anticancer drugs such as doxorubicin (DOX) and increase drug accumulation, thereby improving therapeutic efficacy in tumor cells (73, 74). In the MMP-2/9-responsive delivery system, the nanoparticle carrier does not directly disrupt the ECM; instead, its ECM-modulating effect follows an indirect chain: MMP-2/9 (highly expressed by CAFs) degrades the enzyme-sensitive carrier (gelatin-coated gold nanoparticles modified with RGD peptides), triggering the release of loaded drugs (DOX) (75). The released drugs then inhibit CAF activation/proliferation (e.g., downregulating collagen-synthesis gene COL1A1 in CAFs), reducing CAF-secreted ECM components (collagen, fibronectin) (76). Simultaneously, targeted elimination of CAFs impairs their ECM-remodeling ability, decreasing tumor stromal density, and ultimately achieving indirect ECM modulation. Responsive release system design strategy is illustrated in **Figure 1B** and summarized in **Supplementary Table 2**.

### 3.3 Genetic and metabolic intervention strategies

Nanomaterials can deliver gene drugs or metabolic inhibitors to block the ability of CAFs to promote drug resistance at the molecular level. For example, mPEG-PLGA loaded baicalein can block TGF-β signaling by inhibiting the phosphorylation of key proteins in the TGF-β signaling pathway such as Smad2/3, thereby inhibiting CAF activation (77, 78). Furthermore, baicalein can inhibit CAF migration and invasion by downregulating TGF-β expression and its downstream target genes such as N-cadherin and vimentin (77, 79). SPIONs delivering fibroblast growth factor 2 interferes with TGF-β1-induced CAF activation (68). Nanomaterials target myCAFs through “multivalent recognition-efficient endocytosis”, blocking the communication between CAFs and tumor cells and simultaneously loading gemcitabine and CXCL12 antagonists to block the drug resistance signaling pathway between CAFs and tumor cells (80, 81). The hollow structure of carbon nanotubes is suitable for carrying gene drugs such as siRNAs. Surface modification with hyaluronic acid is performed to target the CD44 receptor on the surface of CAFs; this achieves specific silencing of fibrosis-promoting genes such as COL1A1, thereby improving the delivery of siRNAs and increasing stability and targeting *in vivo* (82).

To interfere with the metabolic coupling between CAFs and tumor cells (such as lactic acid shuttle and glutamine metabolism) and deprive tumor cells of energy sources, PLGA nanoparticles loaded with glutamine inhibitor (CB-839) can target PDGFR-β on the CAF surface, decreasing glutamine secretion and increasing the efficacy of cisplatin in gastric cancer (83, 84). Functional nucleic acid (FNA)-based nanoplatforms combined with ZnO nanoparticles can deplete GSH in the TME, activate ferroptosis, and inhibit P-gp mediated drug efflux, thus overcoming multiple drug resistance (85–90). Genetic and metabolic intervention strategies is illustrated in **Figure 1C** and summarized in **Supplementary Table 3**.

### 3.4 Synergy strategies

Multiple therapeutic approaches can be integrated to synergistically reverse CAF-mediated resistance networks through co-loading or sequential release of nanomaterials. Combination treatments based on chemotherapy targeting CAFs inhibit CAF function and kill tumor cells simultaneously, blocking the protective effect of CAFs on tumor cells. For example, liposomes co-loaded with nilotinib (CAF activation inhibitor) and doxorubicin inhibit the secretion of IL-6 by CAFs, thereby impairing the protective effect of CAFs on tumor cells (91). IL-6 secreted by CAFs can inhibit tumor cell sensitivity to chemotherapy drugs through the JAK/STAT signaling pathway, and nilotinib enhances tumor cell sensitivity to doxorubicin by blocking the corresponding signaling pathway (92). PCL-luteinizing hormone-releasing hormone (LHRH) nanoparticles are a novel drug delivery system capable of loading paclitaxel and the photothermal agent IR780; this system targets ovarian cancer by modifying LHRH peptides. The system utilizes the photothermal effect of near-infrared light to destroy the tumor ECM, which increases drug penetration and release and improves the inhibitory rate in drug-resistant tumors (93). Dual-responsive nanoparticles can release FAP inhibitors in the TME to decrease ECM stiffness, followed by the release of chemotherapeutic drugs such as paclitaxel to kill tumor cells. This strategy is particularly applicable to paclitaxel-resistant ovarian cancer models because it can overcome drug resistance by altering the TME (94, 95).

Immune-targeting CAF combinations aim to reverse the CAF-mediated immunosuppressive microenvironment and enhance immune cell infiltration and function. PD-L1 is expressed on tumor cells and in CAFs, providing a theoretical basis for combined blockade of PD-L1/PD-1 signaling (96). α-PD-L1 antibodies are co-loaded with CAF targeting peptides in nanoparticles to specifically attack tumors and their microenvironments, ultimately improving antitumor immune responses (97). This combination strategy not only enhances CD8^+^T cell infiltration and activation, but also impairs tumor immune escape by inhibiting CAF function (98).

Dual-response nanoparticles (such as pH/enzyme dual response) and multi-drug co-loading with intelligent response synergy mediate the sequential release of CAF inhibitors and chemotherapy drugs to achieve “microenvironment remodeling and tumor killing”. In the liposome-AuNP hybrid system, the photothermal effect of AuNPs (gold nanoparticles) generates local high temperatures under near infrared light irradiation; this softens the tumor matrix and destroys its physical barrier, improving drug release and penetration (99, 100). Liposomes can carry chemotherapeutic drugs and immunomodulators, and drug release is triggered through a photothermal effect. The liposome-AuNP hybrid system is a promising cancer treatment strategy based on different synergy mechanisms that can effectively overcome physical and physiological obstacles in the TME to improve drug delivery and therapeutic effects (101, 102). Superparamagnetic iron oxide nanoparticles (IONPs)-poly (lactic acid-co-glycolic acid)(PLGA) hybrid nanoparticles are a novel nano-drug delivery system that combines magnetic targeting with sustained release of chemotherapeutic drugs; it can enrich in CAF regions and achieve synergistic killing via hyperthermia and chemotherapy (103, 104). PLGA acts as a drug carrier and prolongs the release time of chemotherapeutic drugs; the sustained release decreases the systemic toxicity of drugs (105, 106). With the introduction of IONPs, nanoparticles produce a thermal effect under an applied magnetic field, and the resulting synergistic effect of hyperthermia and chemotherapy improves the killing efficacy in tumor cells (107, 108). The synergistic strategies combining multiple therapeutic approaches is illustrated in **Figure 1D** and summarized in **Supplementary Table 4**.

## 5 Discussion

Targeting CAFs with nanomaterials provides an innovative pathway to reverse tumor drug resistance; however, its clinical translation is limited by a series of challenges. First, because of the heterogeneity of CAFs, identifying a single marker for all drug resistance subgroups is difficult, and there is overlap with normal fibroblasts, resulting in off-target risk. Second, inorganic materials are associated with liver and spleen toxicity; the biocompatibility of the degradation products of organic materials needs to be verified, and targeting ligands may cause immunogenicity. Third, uniformity and stability are difficult to achieve in the large-scale production of nanomaterials; real-time monitoring technology of *in vivo* targeting efficiency is insufficient, and elucidating the mechanisms underlying the effect of combination therapy requires additional study and biomarker guidance.

Among the nanomaterial-based strategies targeting CAFs, stimuli-responsive release systems and multimodal synergy strategies exhibit the most translational promise, and the reasons are as follows. First, unlike surface marker-based precision targeting limited by CAF heterogeneity and non-specific markers, these two strategies adapt to the dynamic TME. Stimuli-responsive systems (e.g., pH/MMP-2/9-sensitive nanoparticles) utilize inherent TME features for controlled drug release, avoiding off-target risks from static markers. Multimodal synergy (e.g., co-loading CAF inhibitors with chemotherapeutics/immunomodulators) remodels the TME while killing tumors, addressing single-target therapy limitations. Second, they align with clinical needs: some stimuli-responsive systems (e.g., pH-sensitive epirubicin micelles NC-6300) have entered Phase I trials (109), and multimodal strategies can combine with existing chemo/immunotherapies, reducing clinical transformation barriers.

The perspective on key hurdles and paths forward: The core bottleneck is not just “insufficient specificity” or “weak translational evidence”, but the overreliance on “static, single-target” thinking in traditional strategies. Future breakthroughs lie in shifting to “dynamic, subtype-specific intervention”. Leveraging single-cell omics to identify subtype markers (e.g., IL-6R for iCAFs) and AI to design “combinatorial marker-recognition” multivalent nanocarriers will solve heterogeneity issues. Meanwhile, using patient-derived organoids instead of animal models to verify targeting efficiency and safety can fill the “translational gap” between preclinical and clinical studies, which is a more debatable yet transformative path than incremental improvements to existing strategies. Only in this way can CAF-targeted nanodrugs be advanced from basic research to clinical application, truly providing a transformative therapeutic approach for overcoming tumor drug resistance.

## Data Availability

The original contributions presented in the study are included in the article/**Supplementary Material**. Further inquiries can be directed to the corresponding authors.
