# Supplementary material for: Advances in the study of reversing tumor drug resistance by targeting cancer-associated fibroblasts with nanomaterials
Source: Front Immunol. 2025 Nov 19;16:1647988. doi: 10.3389/fimmu.2025.1647988 (PMC12672518; doi:10.3389/fimmu.2025.1647988)
Supplement: Supplementary file 1 [file Supplementaryfile1.docx]

**Supplementary Table 1. Precision targeting strategies for CAF surface markers**

| **Nanomaterial Type** | **Targeting Ligand/Action Target** | **Core Mechanism** | **Key Research Findings** | **Reference(s)** |
| --- | --- | --- | --- | --- |
| Liposomes | FAP antibody | Specifically bind to CAFs via FAP antibody, reducing tumor stromal density | Enhance membrane permeability and increase the accumulation of chemotherapeutic drugs | [55] |
| Bismuth ferrite harmonic nanoparticles | FAP antibody | Target CAFs in pancreatic cancer, release chemotherapeutic drugs, and regulate tumor interstitial stiffness | Decrease tumor interstitial stiffness by 40% and increase gemcitabine penetration by 2.5-fold | [56] |
| Chitosan nanoparticles | None (enriched via mucosal adhesion); FAP gene (loaded with FAP siRNA) | Enrich in tumor stroma through mucosal adhesion, deliver FAP siRNA to downregulate FAP expression in CAFs | Inhibit FAP expression in CAFs and block tumor angiogenesis | [57] |
| H-ferritin nanocages | FAP antibody fragments | Target CAFs via FAP antibody fragments, deliver navitoclax | Significantly enhance the cytotoxicity of navitoclax against CAFs | [58] |
| Nanoliposomes | Tenascin C (a protein secreted by CAFs) | Specifically bind to tenascin C secreted by CAFs, deliver navitoclax | Achieve selective apoptosis of CAFs | [59] |
| CRISPR-Cas9 nanovector | CXCR4 gene (in CAFs) | Knock out the CXCR4 gene in CAFs, blocking the CXCL12/CXCR4 signaling axis | Inhibit the CXCL12/CXCR4 axis-mediated maintenance of stem cell stemness and induction of EMT | [60-62] |
| SPIONs | None (act via alternating magnetic fields) | Induce CAF apoptosis under the action of alternating magnetic fields | Effectively inhibit CAF activity and alter the TME | [63-64] |
| Abbreviations：CAFs,cancer-associated fibroblasts; CXCR4,c-x-c chemokine receptor type 4; EMT,epithelial-mesenchymal transition; FAP,fibroblast activation protein; siRNA,small interfering RNA; SPIONs,superparamagnetic iron oxide nanoparticles; TME,tumor microenvironment. | | | | |
