# Supplementary material for: Advances in the study of reversing tumor drug resistance by targeting cancer-associated fibroblasts with nanomaterials
Source: Front Immunol. 2025 Nov 19;16:1647988. doi: 10.3389/fimmu.2025.1647988 (PMC12672518; doi:10.3389/fimmu.2025.1647988)
Supplement: Supplementary file 2 [file Supplementaryfile2.docx]

**Supplementary Table 2. Responsive release systems utilizing TME characteristics for CAF-targeted drug delivery**

| **Nanomaterial Type** | **Stimulus for Release** | **Core Design/Mechanism** | **Key Research Findings** | **Reference(s)** |
| --- | --- | --- | --- | --- |
| pH-sensitive nanoparticles | Acidic nature of the TME | Exploit the acidic TME to enable efficient drug delivery and controlled release | Overcome chemotherapy resistance and reduce systemic side effects | [65] |
| GO-based nanoparticles | Acidic conditions in the TME | Modify the GO surface with pH-sensitive prodrug molecules to achieve controlled release under acidic environments | Facilitate targeted drug release in the CAF-rich microenvironment, supporting cancer therapy | [66] |
| Gold nanoparticles | MMP-2/9, highly expressed in CAFs | Coat with enzyme-degradable carriers (e.g., gelatin modified with RGD peptides) to target CAFs and disrupt the ECM | Enable specific targeting of CAFs and ECM disruption via MMP-2/9-mediated carrier degradation | [67, 68] |
| Gelatin-based nanoparticles | MMP-2/9 ,highly expressed in CAFs | Achieve controlled drug release through degradation by MMP-2/9 in the TME | Specifically degraded in the TME to release loaded anticancer drugs (e.g., DOX), increase intratumoral drug accumulation, and improve therapeutic efficacy on tumor cells | [69, 70] |
| Abbreviations：CAFs,cancer-associated fibroblasts; DOX,doxorubicin; ECM,extracellular matrix; GO,graphene oxide; MMP-2/9,matrix metalloproteinase-2/9; RGD,arginine-glycine-aspartic acid;TME,tumor microenvironment. | | | | |
