# Supplementary material for: Advances in the study of reversing tumor drug resistance by targeting cancer-associated fibroblasts with nanomaterials
Source: Front Immunol. 2025 Nov 19;16:1647988. doi: 10.3389/fimmu.2025.1647988 (PMC12672518; doi:10.3389/fimmu.2025.1647988)
Supplement: Supplementary file 3 [file Supplementaryfile3.docx]

**Supplementary Table 3. Genetic and metabolic intervention strategies for targeting CAFs**

| **Nanomaterial**  **Type** | **Loaded Agent/**  **Functional Molecule** | **Target/**  **Molecular Target** | **Core Mechanism** | **Key Research Findings** | **Reference(s)** |
| --- | --- | --- | --- | --- | --- |
| mPEG-PLGA nanoparticles | Baicalein | TGF-β signaling pathway (Smad2/3, TGF-β, N-cadherin, vimentin) | Inhibit phosphorylation of Smad2/3 (key proteins in TGF-β pathway); downregulate TGF-β expression and its downstream target genes (N-cadherin, vimentin) | Block TGF-β signaling to inhibit CAF activation; reduce CAF migration and invasion | [71, 72, 73] |
| SPIONs | FGF2 | TGF-β1-induced CAF activation | Deliver FGF2 to interfere with the process of CAF activation triggered by TGF-β1 | Suppress TGF-β1-mediated CAF activation | [74] |
| Nanomaterials (unspecified type) | Gemcitabine; CXCL12 antagonists | myCAFs; CAF-tumor cell communication | Target myCAFs via "multivalent recognition-efficient endocytosis"; block CAF-tumor cell communication and CAF-mediated drug resistance signaling pathways | Inhibit crosstalk between CAFs and tumor cells; block drug resistance signaling axes | [75, 76] |
| Carbon nanotubes | siRNAs (fibrosis-promoting genes, e.g., COL1A1) | CD44 receptor (on CAFs); COL1A1 gene | Modify surface with hyaluronic acid to target CD44 on CAFs; deliver siRNAs to specifically silence fibrosis-promoting genes (e.g., COL1A1) | Improve siRNA delivery efficiency; enhance in vivo stability and targeting ability; silence COL1A1 to inhibit fibrosis | [77] |
| PLGA nanoparticles | Glutamine inhibitor (CB-839) | PDGFR-β (on CAFs); CAF-tumor cell metabolic coupling (glutamine metabolism) | Target PDGFR-β on CAFs to deliver CB-839; inhibit glutamine secretion by CAFs to disrupt CAF-tumor cell metabolic coupling | Reduce glutamine supply to tumor cells; enhance the efficacy of cisplatin in gastric cancer | [78, 79] |
| FNA-based nanoplatforms | FNAs; ZnO nanoparticles | TME (GSH); P-gp (drug efflux pump) | Combine with ZnO nanoparticles to deplete GSH in TME; activate ferroptosis; inhibit P-gp-mediated drug efflux | Overcome multiple drug resistance by regulating TME redox status, inducing ferroptosis, and blocking drug efflux | [80-85] |
| Abbreviations：CAFs,cancer-associated fibroblasts; CD44, cluster of differentiation 44;COL1A1,collagen type I alpha 1 chain; CXCL12,c-x-c chemokine ligand 12; FGF2,fibroblast growth factor 2; FNA,functional nucleic acid; GSH,glutathione; mPEG-PLGA,methoxy-Poly(Ethylene Glycol)-Poly(Lactic-co-Glycolic Acid); myCAFs,myofibroblastic cancer-associated fibroblasts; PDGFR-β,platelet-derived growth factor receptor-β; P-gp,p-glycoprotein; PLGA,poly(Lactic-co-Glycolic Acid); siRNA,small interfering RNA; SPIONs,superparamagnetic iron oxide nanoparticles; TGF-β,transforming growth factor-β; TME,tumor microenvironment. | | | | | |
