# Supplementary material for: Advances in the study of reversing tumor drug resistance by targeting cancer-associated fibroblasts with nanomaterials
Source: Front Immunol. 2025 Nov 19;16:1647988. doi: 10.3389/fimmu.2025.1647988 (PMC12672518; doi:10.3389/fimmu.2025.1647988)
Supplement: Supplementary file 4 [file Supplementaryfile4.docx]

**Supplementary Table 4. Synergy strategies for targeting CAFs to reverse tumor drug resistance**

| **Nanomaterial**  **Type** | **Loaded Agent/**  **Functional Component** | **Target/**  **Application Context** | **Core Synergy Mechanism** | **Key**  **Research Findings** | **Reference(s)** |
| --- | --- | --- | --- | --- | --- |
| Liposomes | Nilotinib (CAF activation inhibitor); Doxorubicin (chemotherapeutic) | CAFs; Tumor cells (general cancer context) | Co-load nilotinib and doxorubicin: Nilotinib inhibits CAF-secreted IL-6 and blocks JAK/STAT signaling, while doxorubicin kills tumor cells | Impair CAFs’ protective effect on tumor cells; enhance tumor cell sensitivity to doxorubicin by blocking IL-6/JAK/STAT pathway | [86, 87] |
| PCL-LHRH nanoparticles | Paclitaxel (chemotherapeutic); IR780 (photothermal agent) | Ovarian cancer (drug-resistant models) | Modify with LHRH peptides for ovarian cancer targeting; IR780 exerts photothermal effect to destroy tumor ECM, while paclitaxel kills tumor cells | Increase drug penetration and release via ECM destruction; improve inhibitory rate in drug-resistant ovarian tumors | [88] |
| Dual-responsive nanoparticles | FAP inhibitors; Paclitaxel (chemotherapeutic) | Paclitaxel-resistant ovarian cancer | Sequential release: First release FAP inhibitors to decrease ECM stiffness, then release paclitaxel to kill tumor cells | Overcome paclitaxel resistance by remodeling the TME (reducing ECM stiffness) | [89, 90] |
| Nanoparticles (unspecified type) | α-PD-L1 antibodies; CAF-targeting peptides | Tumors with CAF-mediated immunosuppression | Co-load α-PD-L1 antibodies and CAF-targeting peptides: Target CAFs and block PD-L1/PD-1 signaling simultaneously | Reverse CAF-mediated immunosuppressive microenvironment; enhance CD8+ T cell infiltration/activation; impair tumor immune escape | [91, 92, 93] |
| Liposome-AuNP hybrid system | Chemotherapeutic drugs; Immunomodulators | Tumors with TME physical/physiological obstacles | AuNPs generate local hyperthermia under near-infrared light to soften tumor matrix (destroy physical barriers); liposomes deliver drugs/immunomodulators, with release triggered by photothermal effect | Overcome TME physical/physiological obstacles; improve drug delivery efficiency and therapeutic effects via photothermal-drug-immunomodulation synergy | [94, 95, 96, 97] |
| Superparamagnetic IONPs-PLGA hybrid nanoparticles | Chemotherapeutic drugs | CAF-rich tumor regions | Combine magnetic targeting (IONPs) for CAF region enrichment, PLGA-mediated sustained drug release, and IONP-induced hyperthermia (under magnetic field) | Achieve synergistic tumor killing via hyperthermia and chemotherapy; prolong drug release (reduce systemic toxicity) and enrich in CAF regions | [98, 99, 100, 101, 102, 103] |
| Abbreviations：α-PD-L1,α-Programmed Death-Ligand 1; AuNPs,gold nanoparticles; CAFs,cancer-associated fibroblasts; ECM,extracellular matrix; FAP,fibroblast activation protein; IL-6,interleukin-6; IONPs,iron oxide nanoparticles; JAK/STAT,janus kinase/signal transducer and activator of transcription; LHRH,luteinizing hormone-releasing hormone; PD-1,programmed death-1; PCL,polycaprolactone; PLGA,poly(Lactic-co-Glycolic Acid); TME,tumor microenvironment. | | | | | |
